# Supplementary material for: Kribbellichelins A and B, Two New Antibiotics from Kribbella sp. CA-293567 with Activity against Several Human Pathogens
Source: Molecules. 2022 Sep 26;27(19):6355. doi: 10.3390/molecules27196355 (PMC9570599; doi:10.3390/molecules27196355)
Supplement: Supplementary file 1 [file molecules-27-06355-s001.zip › molecules-1871121-supplementary.pdf]

Supplementary Materials

Kribbellichelins A and B, Two New Antibiotics from *Kribbella* sp. CA-293567 with Activity against Several Human Pathogens

Jorge R. Virués-Segovia, Fernando Reyes, Sandra Ruíz, Jesús Martín, Ignacio Fernández-Pastor, Carlos Justicia, Mercedes de la Cruz, Caridad Díaz, Thomas A. Mackenzie, Olga Genilloud, Ignacio González and José R. Tormo *

Fundación MEDINA, Av. Conocimiento 34, Health Sciences Technology Park, 18016 Granada, Spain

* Correspondence: ruben.tormo@medinaandalucia.es (J.R.T.); Tel.: +34-958-993-965

Table S1. Taxonomical classification of the 108 actinomycetes strains isolated from the aerial part, roots and rhizosphere of the endemic plant *Limonium majus* across orders, families and genus. In bold is highlighted the taxonomical classification of the *Kribbella* strain studied in detail.

Tax Order	Family	Genus	Number	Origin
Corynebacteriales	*Nocardiaceae*	*Nocardia*	1	Rhizosphere
Micrococcales	*Brevibacteriaceae*	*Brevibacterium*	3	Roots (n =1) and rhizosphere (n=2)
	Microbacteriaceae	*Microbacterium*	2	Roots and rhizosphere
		Agrococcus	2	Rhizosphere
		Curtobacterium	1	Roots
	Micrococcaceae	*Kocuria*	2	Aerial part
		Micrococcus	1	Aerial part
		Arthrobacter	2	Roots
		Pseudarthrobacter	1	Rhizosphere
	Promicromonosporaceae	*Promicromonospora*	3	Rhizosphere
		Isoptericola	1	Rhizosphere
	Dermabacteraceae	*Brachybacterium*	1	Rhizosphere
Micromonosporales	*Micromonosporaceae*	*Micromonospora*	17	Roots (n=3) and rhizosphere (n=10)
		Xiangella	1	Rhizosphere
Mycobacteriales	*Mycobacteriaceae*	*Mycolicibacterium*	1	Rizhosphere
Propionibacteriales	***Nocardioidaceae***	*Aeromicrobium*	1	Rhizosphere
		Kribbella	2	Rhizosphere
		Nocardioides	4	Rhizosphere
	Propionibacteriaceae	*Friedmanniella*	1	Roots
Pseudonocardiales	*Pseudonocardiaceae*	*Saccharopolyspora*	1	Roots
		Actinokineospora	2	Rhizosphere
Streptomycetales	*Streptomycetaceae*	*Streptomyces*	47	Aerial part (n=5), roots (n=7) and rhizosphere (n=35)
Streptosporangiales	*Streptosporangiaceae*	*Nonomuraea*	1	Rhizosphere
Frankiales	*Geodermatophilaceae*	*Modestobacter*	1	Aerial part
Kineosporiales	*Kineosporiaceae*	*Angustibacter*	1	Aerial part
Incertae sedis			8	Roots (n=5) and rhizosphere (n=3)
Total			108	Aerial part (n=10), roots (n=26) and rhizosphere (n=72)

Table S2. Taxonomical classification of the 96 actinomycetes classified based on their ribosomal 16S rDNA. Individual GenBank accession numbers indicated.

Strain Name	Accession Number	Phylum	Class	Order	Family	Genus	Species
CA-289638	OP442265	Actinomycetota	Actinomycetes	Streptomycetales	Streptomycetaceae	Streptomyces	finlayi
CA-289639	OP442266	Actinomycetota	Actinomycetes	Streptomycetales	Streptomycetaceae	Streptomyces	chumphonensis
CA-289640	OP442267	Actinomycetota	Actinomycetes	Streptomycetales	Streptomycetaceae	Streptomyces	bacillaris
CA-289641	OP442268	Actinomycetota	Actinomycetes	Streptomycetales	Streptomycetaceae	Streptomyces	bacillaris
CA-289642	OP442269	Actinomycetota	Actinomycetes	Streptomycetales	Streptomycetaceae	Streptomyces	specialis
CA-290269	OP442270	Actinomycetota	Actinomycetes	Micromonosporales	Micromonosporaceae	Micromonospora	vinacea
CA-290270	OP442271	Actinomycetota	Actinomycetes	Micromonosporales	Micromonosporaceae	Xiangella	phaseoli
CA-290272	OP442272	Actinomycetota	Actinomycetes	Streptomycetales	Streptomycetaceae	Streptomyces	cacaoi
CA-290273	OP442273	Actinomycetota	Actinomycetes	Micromonosporales	Micromonosporaceae	Micromonospora	vinacea
CA-290274	OP442274	Actinomycetota	Actinomycetes	Streptomycetales	Streptomycetaceae	Streptomyces	carpaticus
CA-290275	OP442275	Actinomycetota	Actinomycetes	Streptomycetales	Streptomycetaceae	Streptomyces	carpaticus
CA-290555	OP442276	Actinomycetota	Actinomycetes	Pseudonocardiales	Pseudonocardiaceae	Saccharopolyspora	gloriosae
CA-290924	OP442277	Actinomycetota	Actinomycetes	Micromonosporales	Micromonosporaceae	Micromonospora	vinacea
CA-290925	OP442278	Actinomycetota	Actinomycetes	Streptomycetales	Streptomycetaceae	Streptomyces	olivaceus
CA-291033	OP442279	Actinomycetota	Actinomycetes	Streptomycetales	Streptomycetaceae	Streptomyces	chumphonensis
CA-291038	OP442280	Actinomycetota	Actinomycetes	Streptomycetales	Streptomycetaceae	Streptomyces	ambofaciens
CA-291078	OP442281	Actinomycetota	Actinomycetes	Streptomycetales	Streptomycetaceae	Streptomyces	harbinensis
CA-293550	OP442282	Actinomycetota	Actinomycetes	Propionibacteriales	Kribbellaceae	Kribbella	deserti
CA-293551	OP442283	Actinomycetota	Actinomycetes	Streptomycetales	Streptomycetaceae	Streptomyces	pratensis
CA-293552	OP442284	Actinomycetota	Actinomycetes	Micrococcales	Promicromonosporaceae	Promicromonospora	alba
CA-293553	OP442285	Actinomycetota	Actinomycetes	Streptomycetales	Streptomycetaceae	Streptomyces	pratensis
CA-293555	OP442286	Actinomycetota	Actinomycetes	Streptomycetales	Streptomycetaceae	Streptomyces	galilaeus
CA-293556	OP442287	Actinomycetota	Actinomycetes	Streptomycetales	Streptomycetaceae	Streptomyces	pratensis
CA-293557	OP442288	Actinomycetota	Actinomycetes	Streptomycetales	Streptomycetaceae	Streptomyces	galilaeus
CA-293558	OP442289	Actinomycetota	Actinomycetes	Streptomycetales	Streptomycetaceae	Streptomyces	peucetius

CA-293559	OP442290	Actinomycetota	Actinomycetes	Micromonosporales	Micromonosporaceae	Micromonospora	saelicesensis
CA-293560	OP442291	Actinomycetota	Actinomycetes	Mycobacteriales	Nocardiaceae	Nocardia	sungurluensis
CA-293561	OP442292	Actinomycetota	Actinomycetes	Micromonosporales	Micromonosporaceae	Micromonospora	vinacea
CA-293562	OP442293	Actinomycetota	Actinomycetes	Streptomycetales	Streptomycetaceae	Streptomyces	cinereus
CA-293563	OP442294	Actinomycetota	Actinomycetes	Streptomycetales	Streptomycetaceae	Streptomyces	sparsus
CA-293564	OP442295	Actinomycetota	Actinomycetes	Streptomycetales	Streptomycetaceae	Streptomyces	cinereus
CA-293565	OP442296	Actinomycetota	Actinomycetes	Streptomycetales	Streptomycetaceae	Streptomyces	cinereus
CA-293566	OP442297	Actinomycetota	Actinomycetes	Streptomycetales	Streptomycetaceae	Streptomyces	cinereus
CA-293567	OP442298	Actinomycetota	Actinomycetes	Propionibacteriales	Kribbellaceae	Kribbella	ginsengisoli
CA-293568	OP442299	Actinomycetota	Actinomycetes	Streptomycetales	Streptomycetaceae	Streptomyces	sparsus
CA-293569	OP442300	Actinomycetota	Actinomycetes	Streptomycetales	Streptomycetaceae	Streptomyces	ambofaciens
CA-293570	OP442301	Actinomycetota	Actinomycetes	Micromonosporales	Micromonosporaceae	Micromonospora	vinacea
CA-293571	OP442302	Actinomycetota	Actinomycetes	Streptomycetales	Streptomycetaceae	Streptomyces	fulvissimus
CA-293572	OP442303	Actinomycetota	Actinomycetes	Streptomycetales	Streptomycetaceae	Streptomyces	galilaeus
CA-293573	OP442304	Actinomycetota	Actinomycetes	Streptomycetales	Streptomycetaceae	Streptomyces	galilaeus
CA-293574	OP442305	Actinomycetota	Actinomycetes	Micrococcales	Promicromonosporaceae	Promicromonospora	alba
CA-293575	OP442306	Actinomycetota	Actinomycetes	Streptomycetales	Streptomycetaceae	Streptomyces	laurentii
CA-293576	OP442307	Actinomycetota	Actinomycetes	Streptomycetales	Streptomycetaceae	Streptomyces	sampsonii
CA-293577	OP442308	Actinomycetota	Actinomycetes	Streptomycetales	Streptomycetaceae	Streptomyces	pratensis
CA-293579	OP442309	Actinomycetota	Actinomycetes	Streptomycetales	Streptomycetaceae	Streptomyces	laurentii
CA-293580	OP442310	Actinomycetota	Actinomycetes	Pseudonocardiales	Pseudonocardiaceae	Actinokineospora	acnipugnans
CA-293581	OP442311	Actinomycetota	Actinomycetes	Pseudonocardiales	Pseudonocardiaceae	Actinokineospora	acnipugnans
CA-293582	OP442312	Actinomycetota	Actinomycetes	Streptomycetales	Streptomycetaceae	Streptomyces	pratensis
CA-293583	OP442313	Actinomycetota	Actinomycetes	Streptomycetales	Streptomycetaceae	Streptomyces	pratensis
CA-293584	OP442314	Actinomycetota	Actinomycetes	Streptomycetales	Streptomycetaceae	Streptomyces	sampsonii
CA-293585	OP442315	Actinomycetota	Actinomycetes	Micromonosporales	Micromonosporaceae	Micromonospora	profundi
CA-293586	OP442316	Actinomycetota	Actinomycetes	Micromonosporales	Micromonosporaceae	Micromonospora	profundi
CA-293587	OP442317	Actinomycetota	Actinomycetes	Micromonosporales	Micromonosporaceae	Micromonospora	profundi
CA-293588	OP442318	Actinomycetota	Actinomycetes	Streptomycetales	Streptomycetaceae	Streptomyces	laurentii

CA-293589	OP442319	Actinomycetota	Actinomycetes	Streptomycetales	Streptomycetaceae	Streptomyces	puniceus
CA-293590	OP442320	Actinomycetota	Actinomycetes	Micrococcales	Promicromonosporaceae	Promicromonospora	iranensis
CA-293591	OP442321	Actinomycetota	Actinomycetes	Streptomycetales	Streptomycetaceae	Streptomyces	arcticus
CA-293592	OP442322	Actinomycetota	Actinomycetes	Streptomycetales	Streptomycetaceae	Streptomyces	arcticus
CA-293593	OP442323	Actinomycetota	Actinomycetes	Streptomycetales	Streptomycetaceae	Streptomyces	arcticus
CA-293594	OP442324	Actinomycetota	Actinomycetes	Streptomycetales	Streptomycetaceae	Streptomyces	armeniacus
CA-293595	OP442325	Actinomycetota	Actinomycetes	Streptomycetales	Streptomycetaceae	Streptomyces	lunaelactis
CA-294218	OP442326	Actinomycetota	Actinomycetes	Micromonosporales	Micromonosporaceae	Micromonospora	vinacea
CA-294219	OP442327	Actinomycetota	Actinomycetes	Micromonosporales	Micromonosporaceae	Micromonospora	vinacea
CA-294220	OP442328	Actinomycetota	Actinomycetes	Streptomycetales	Streptomycetaceae	Streptomyces	shaanxiensis
CA-294221	OP442329	Actinomycetota	Actinomycetes	Streptomycetales	Streptomycetaceae	Streptomyces	arcticus
CA-294223	OP442330	Actinomycetota	Actinomycetes	Streptosporangiales	Streptosporangiaceae	Nonomuraea	glycinis
CA-294838	OP442331	Actinomycetota	Actinomycetes	Micromonosporales	Micromonosporaceae	Micromonospora	vinacea
CA-294839	OP442332	Actinomycetota	Actinomycetes	Streptomycetales	Streptomycetaceae	Streptomyces	diastatochromogenes
CA-294840	OP442333	Actinomycetota	Actinomycetes	Micromonosporales	Micromonosporaceae	Micromonospora	vinacea
CA-294842	OP442334	Actinomycetota	Actinomycetes	Streptomycetales	Streptomycetaceae	Streptomyces	sampsonii
CA-294843	OP442335	Actinomycetota	Actinomycetes	Micromonosporales	Micromonosporaceae	Micromonospora	vinacea
CB-290381	OP442336	Actinomycetota	Actinomycetes	Micrococcales	Micrococcaceae	Micrococcus	yunnanensis
CB-290382	OP442337	Actinomycetota	Actinomycetes	Micrococcales	Micrococcaceae	Kocuria	rosea
CB-290384	OP442338	Actinomycetota	Actinomycetes	Geodermatophilales	Geodermatophilaceae	Modestobacter	caceresii
CB-290385	OP442339	Actinomycetota	Actinomycetes	Kineosporiales	Kineosporiaceae	Angustibacter	speluncae
CB-290386	OP442340	Actinomycetota	Actinomycetes	Micrococcales	Micrococcaceae	Kocuria	rosea
CB-290525	OP442341	Actinomycetota	Actinomycetes	Micrococcales	Brevibacteriaceae	Brevibacterium	casei
CB-290530	OP442342	Actinomycetota	Actinomycetes	Micrococcales	Micrococcaceae	Arthrobacter	subterraneus
CB-290531	OP442343	Actinomycetota	Actinomycetes	Micrococcales	Microbacteriaceae	Curtobacterium	oceanosedimentum
CB-290532	OP442344	Actinomycetota	Actinomycetes	Micrococcales	Micrococcaceae	Arthrobacter	agilis
CB-290540	OP442345	Actinomycetota	Actinomycetes	Propionibacteriales	Propionibacteriaceae	Friedmanniella	lacustris
CB-291208	OP442346	Actinomycetota	Actinomycetes	Micrococcales	Microbacteriaceae	Microbacterium	oleivorans
CB-294255	OP442347	Actinomycetota	Actinomycetes	Propionibacteriales	Nocardioidaceae	Nocardioides	zeicaulis

CB-294260	OP442348	Actinomycetota	Actinomycetes	Propionibacteriales	Nocardioidaceae	Aeromicrobium	choanae
CB-294262	OP442349	Actinomycetota	Actinomycetes	Micrococcales	Micrococcaceae	Pseudarthrobacter	oxydans
CB-294264	OP442350	Actinomycetota	Actinomycetes	Micrococcales	Microbacteriaceae	Microbacterium	yannicii
CB-294268	OP442351	Actinomycetota	Actinomycetes	Propionibacteriales	Nocardioidaceae	Nocardioides	dokdonensis
CB-294451	OP442352	Actinomycetota	Actinomycetes	Propionibacteriales	Nocardioidaceae	Nocardioides	glacieisoli
CB-294452	OP442353	Actinomycetota	Actinomycetes	Micrococcales	Microbacteriaceae	Agrococcus	citreus
CB-294457	OP442354	Actinomycetota	Actinomycetes	Mycobacteriales	Mycobacteriaceae	Mycolicibacterium	insubricum
CB-294458	OP442355	Actinomycetota	Actinomycetes	Micrococcales	Dermabacteraceae	Brachybacterium	phenoliresistens
CB-294462	OP442356	Actinomycetota	Actinomycetes	Micrococcales	Brevibacteriaceae	Brevibacterium	frigoritolerans
CB-294466	OP442357	Actinomycetota	Actinomycetes	Micrococcales	Promicromonosporaceae	Isoptericola	chiayiensis
CB-294467	OP442358	Actinomycetota	Actinomycetes	Propionibacteriales	Nocardioidaceae	Nocardioides	furvisabuli
CB-294484	OP442359	Actinomycetota	Actinomycetes	Micrococcales	Brevibacteriaceae	Brevibacterium	frigoritolerans
CB-294486	OP442360	Actinomycetota	Actinomycetes	Micrococcales	Microbacteriaceae	Agrococcus	citreus

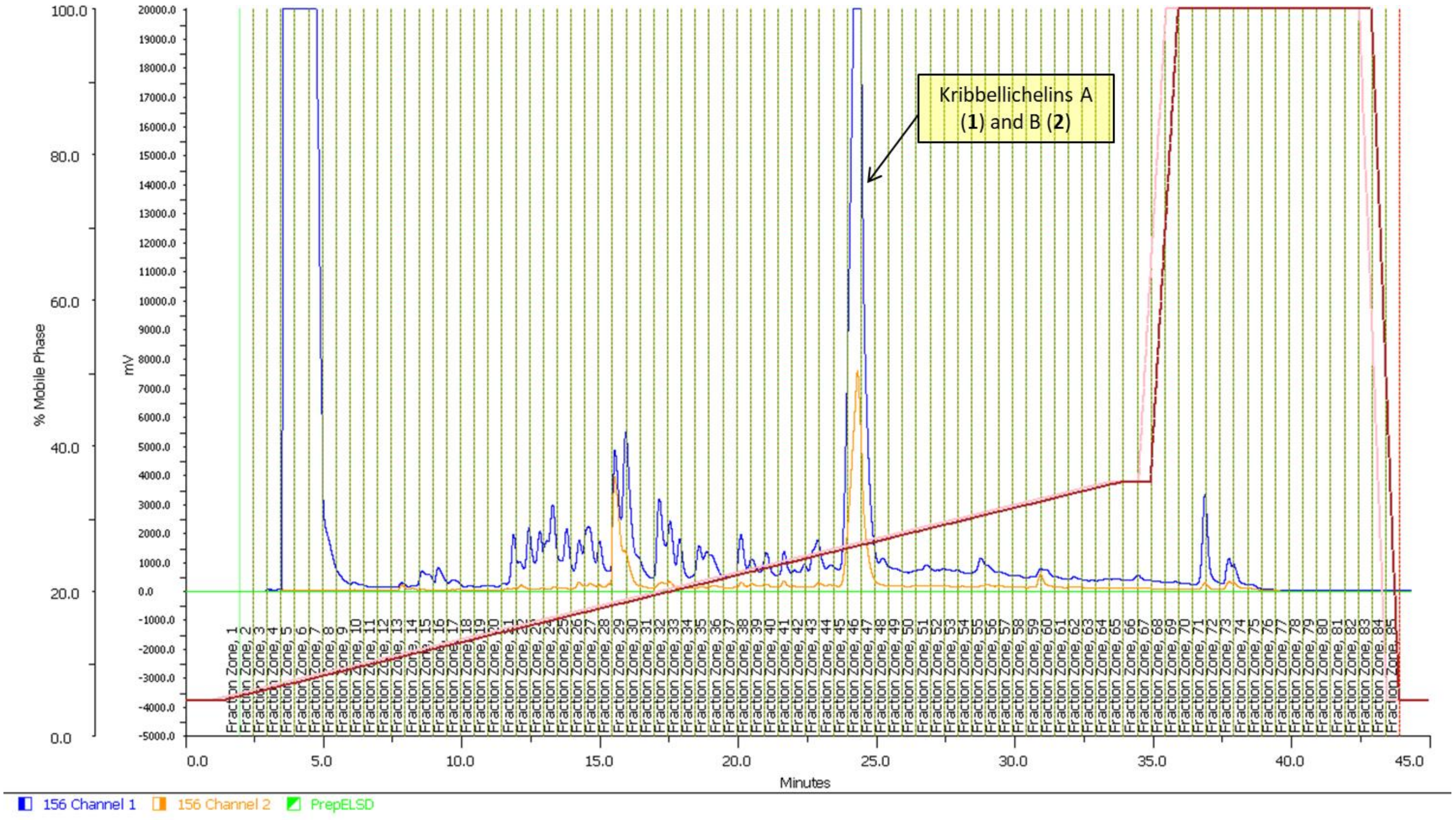

Figure S1. LC-UV (210, orange, and 280, blue trace, nm) chromatogram of fraction FS013 purification by preparative RP-HPLC applying a linear H_2O:CH_3CN gradient (5% CH_3CN: 0-1 min, 5-35%: 1-34 min, 35%: 34-35 min, 35-100%: 35-36 min, 100%: 36-43 min). Both solvents contain 0.1% TFA.

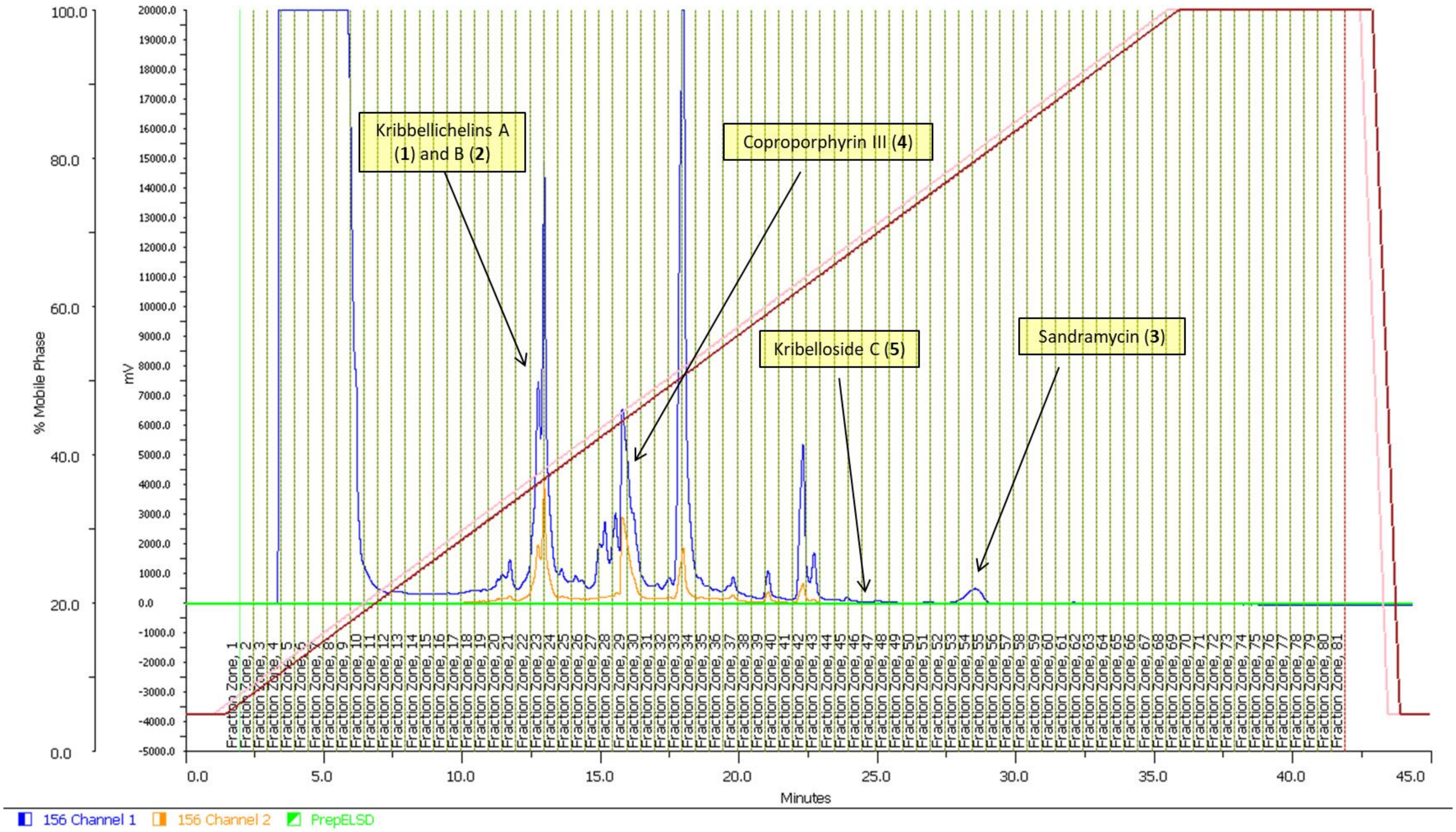

Figure S2. LC-UV (210, orange, and 280, blue trace, nm) chromatogram of FS018 purification by preparative RP-HPLC applying a linear H_2O:CH_3CN gradient (5% CH_3CN: 0-1 min, 5-100%: 1-36 min, 100%: 36-43 min). Both solvents contain 0.1% TFA.

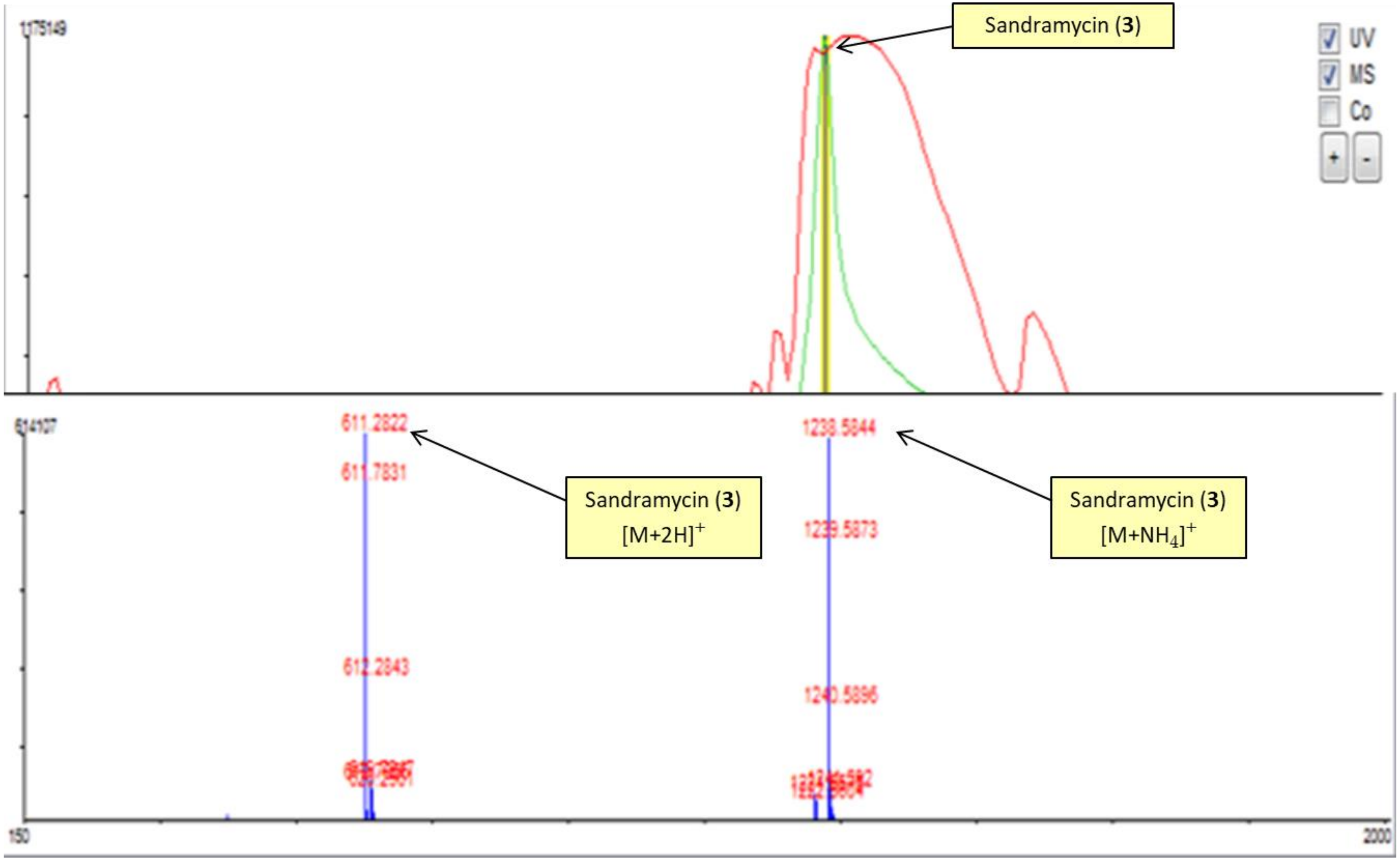

Figure S3. LC-HRMS ((+)-ESI-TOF) analysis of FS018 fractions containing compound **3**.

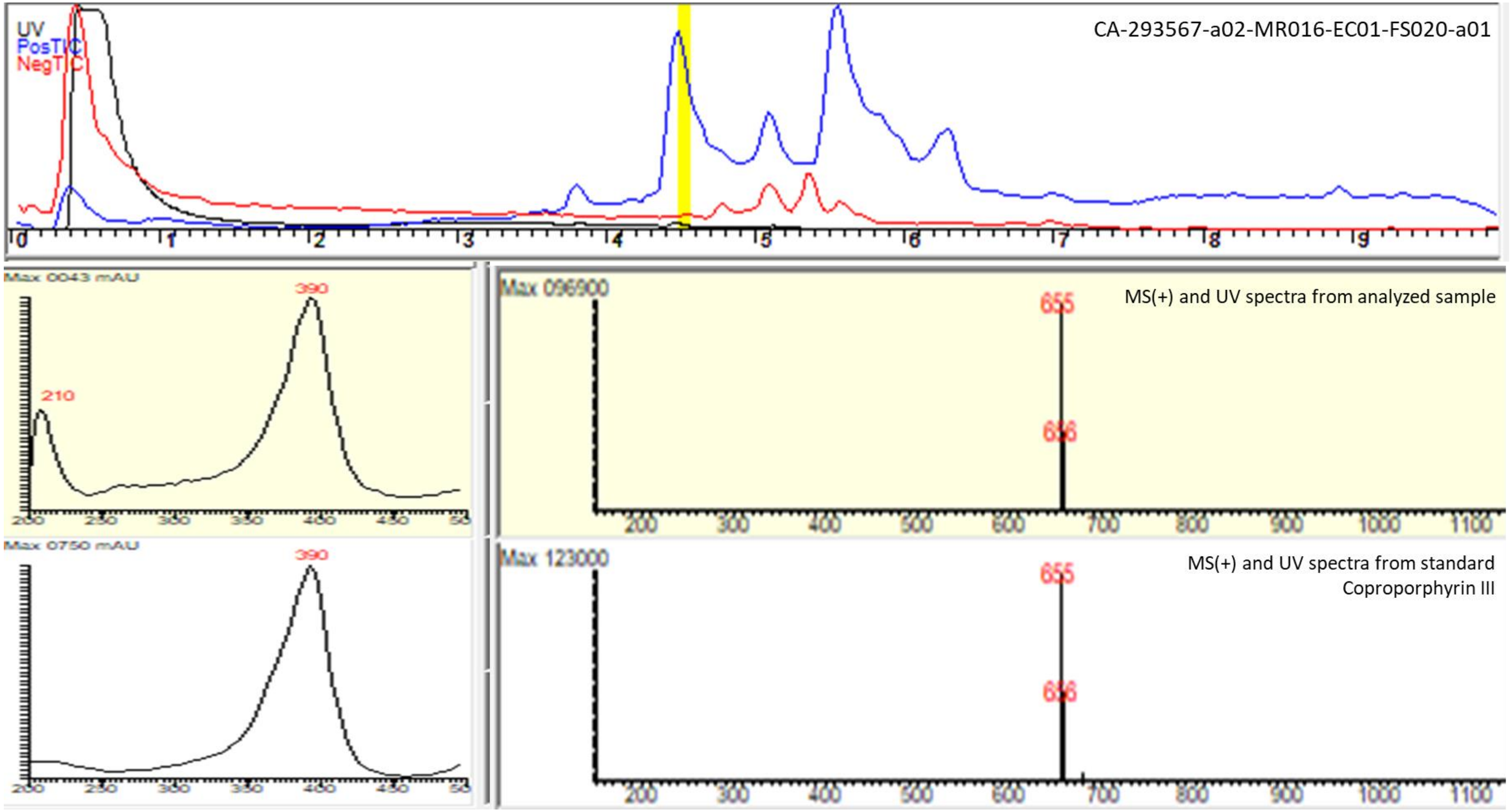

Figure S4. LC-HRMS ((+)-ESI-TOF) analysis of fractions containing compound **4**.

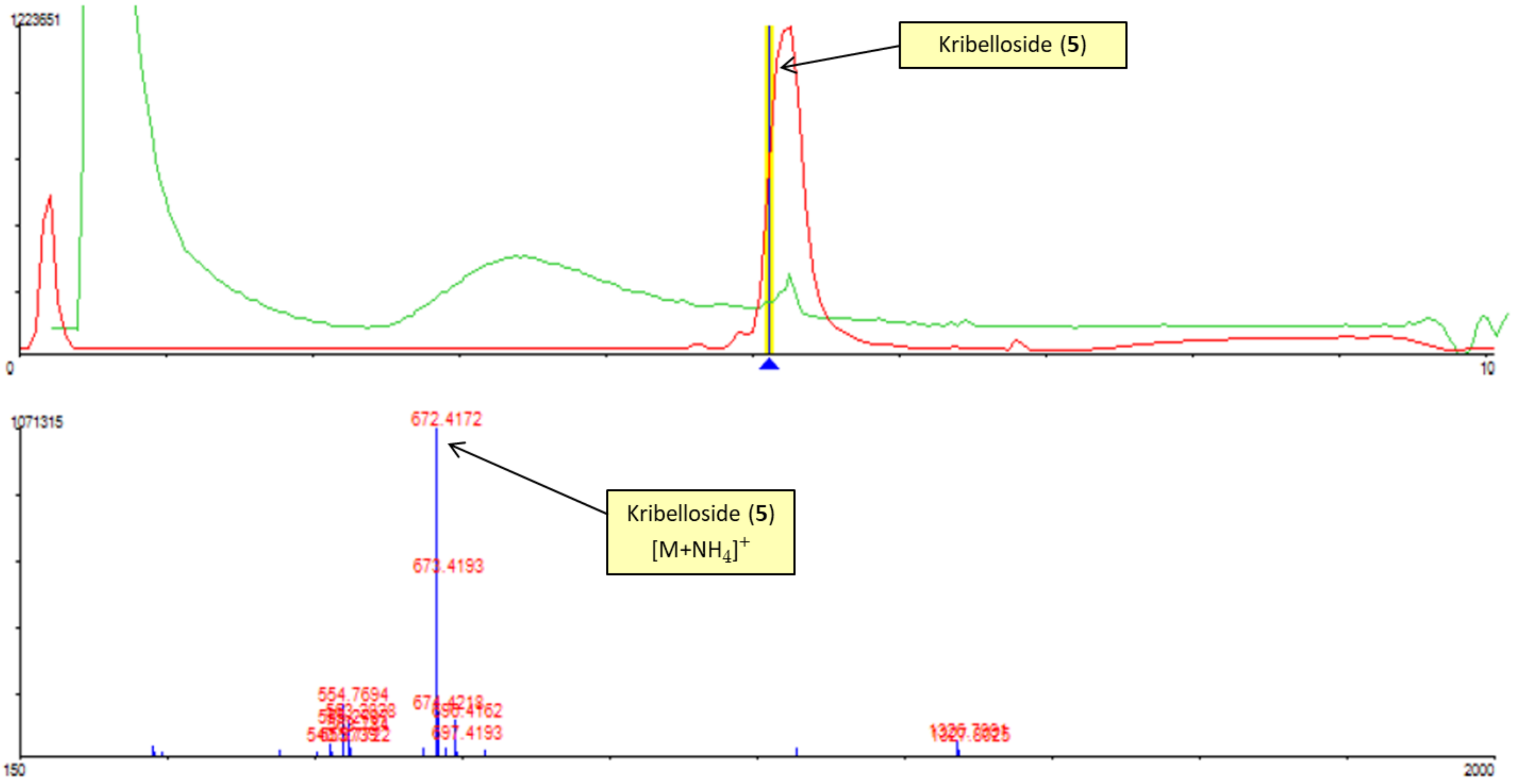

Figure S5. LC-HRMS ((+)-ESI-TOF) analysis of FS018 fractions containing compound **5**.

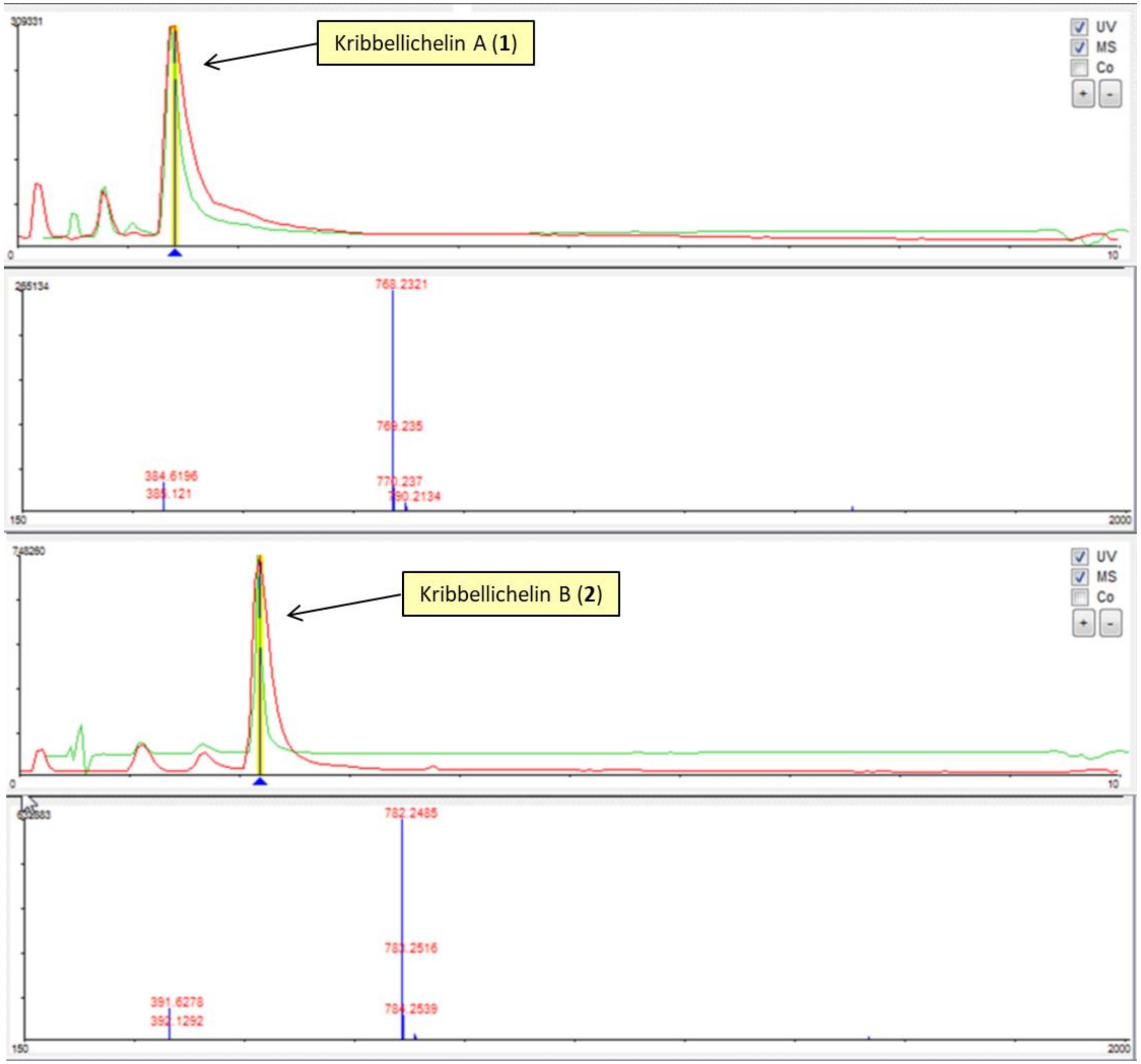

Figure S6. LC-HRMS ((+)-ESI-TOF) analysis of fractions containing compounds **1** and **2**.

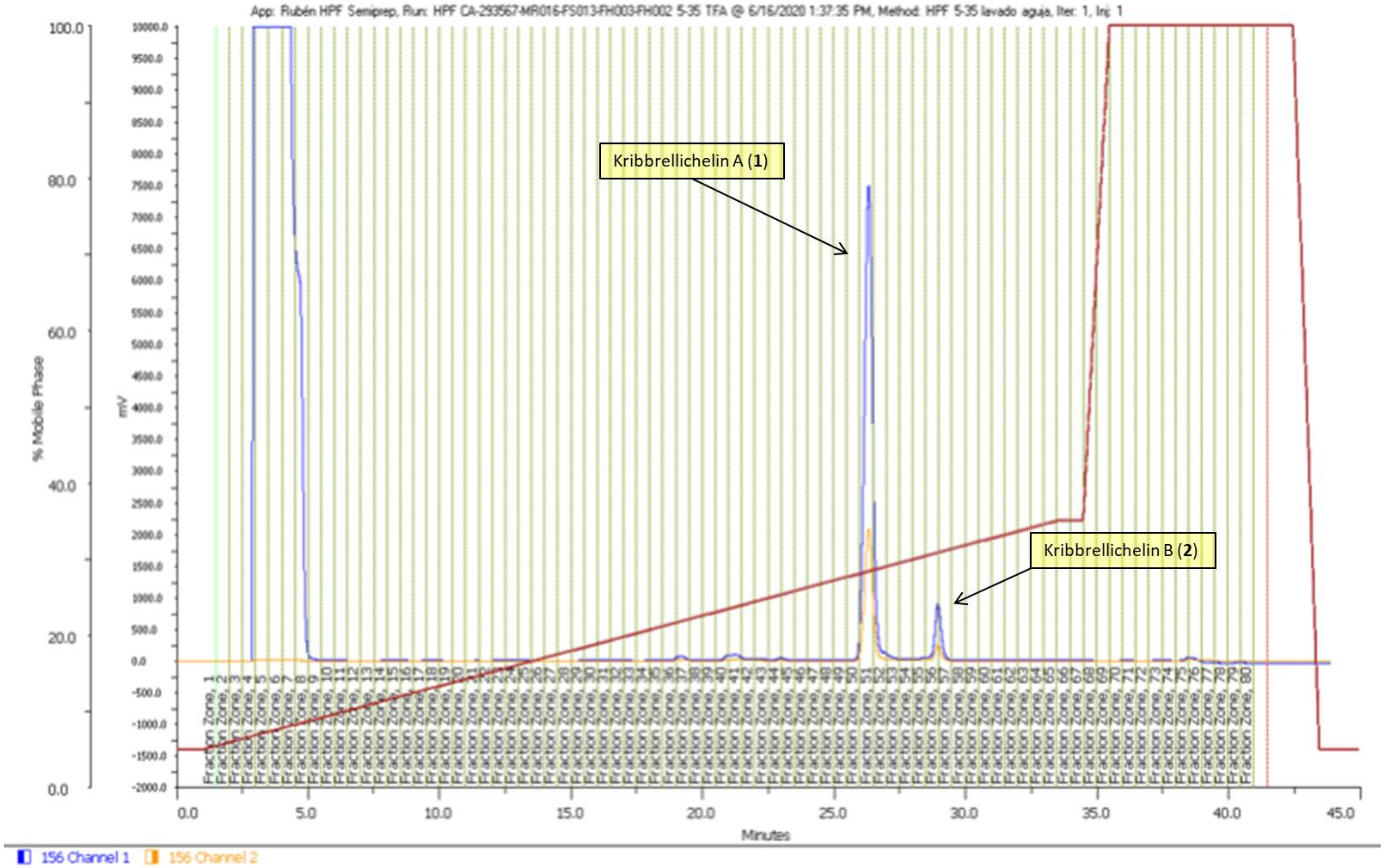

Figure S7. LC-UV (210, orange, and 280, blue trace, nm) chromatogram of the purification of fractions containing compounds **1** and **2** by semipreparative RP-HPLC applying a linear H_2O:CH_3CN gradient (5% CH_3CN: 0-1 min, 5-100%: 1-36 min, 100%: 36-43 min). Both solvents contain 0.1% TFA.

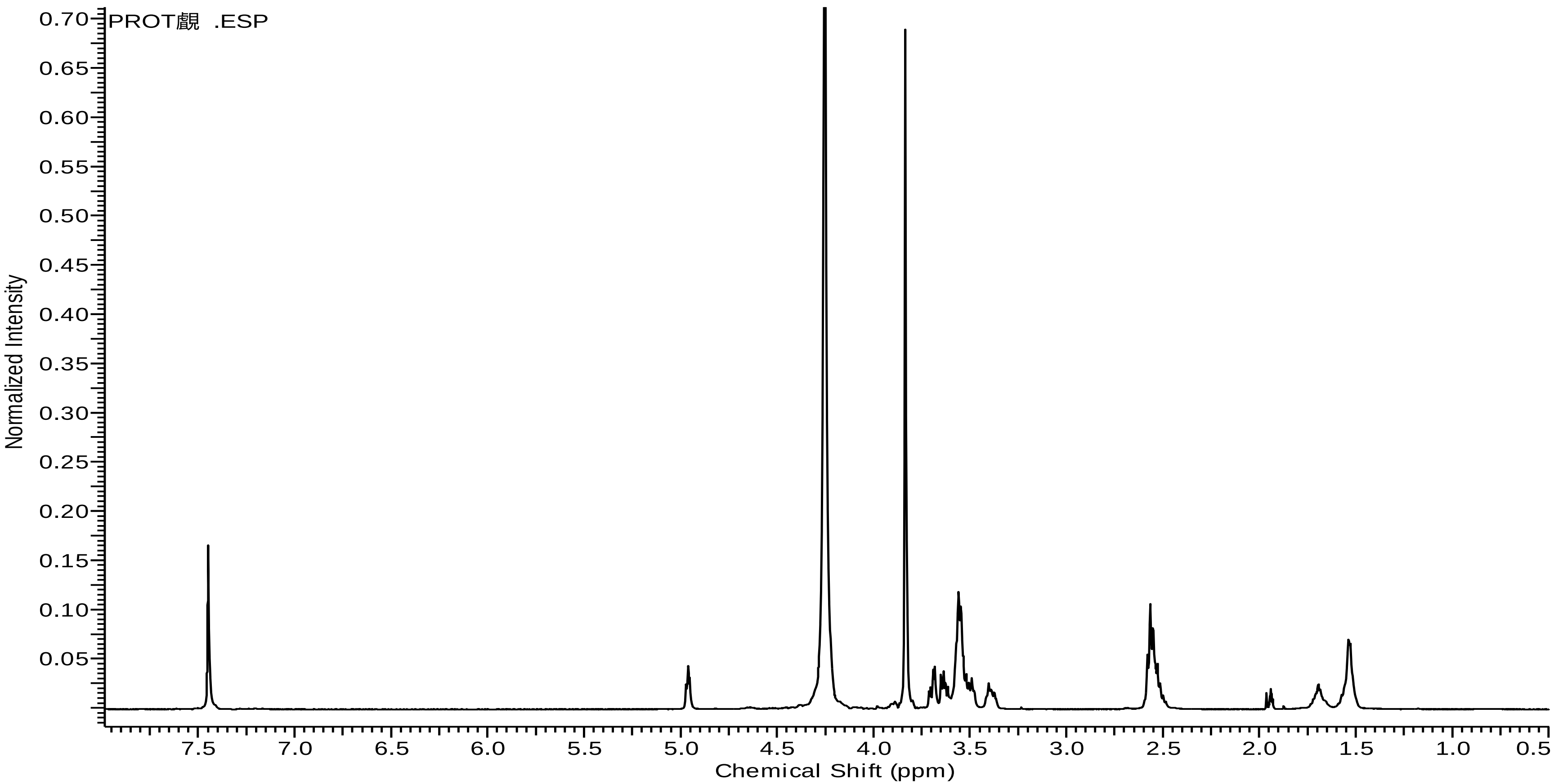

Figure S8. ^{1}H NMR spectrum of compound **1** (CD_3CN/D_2O 1:1).

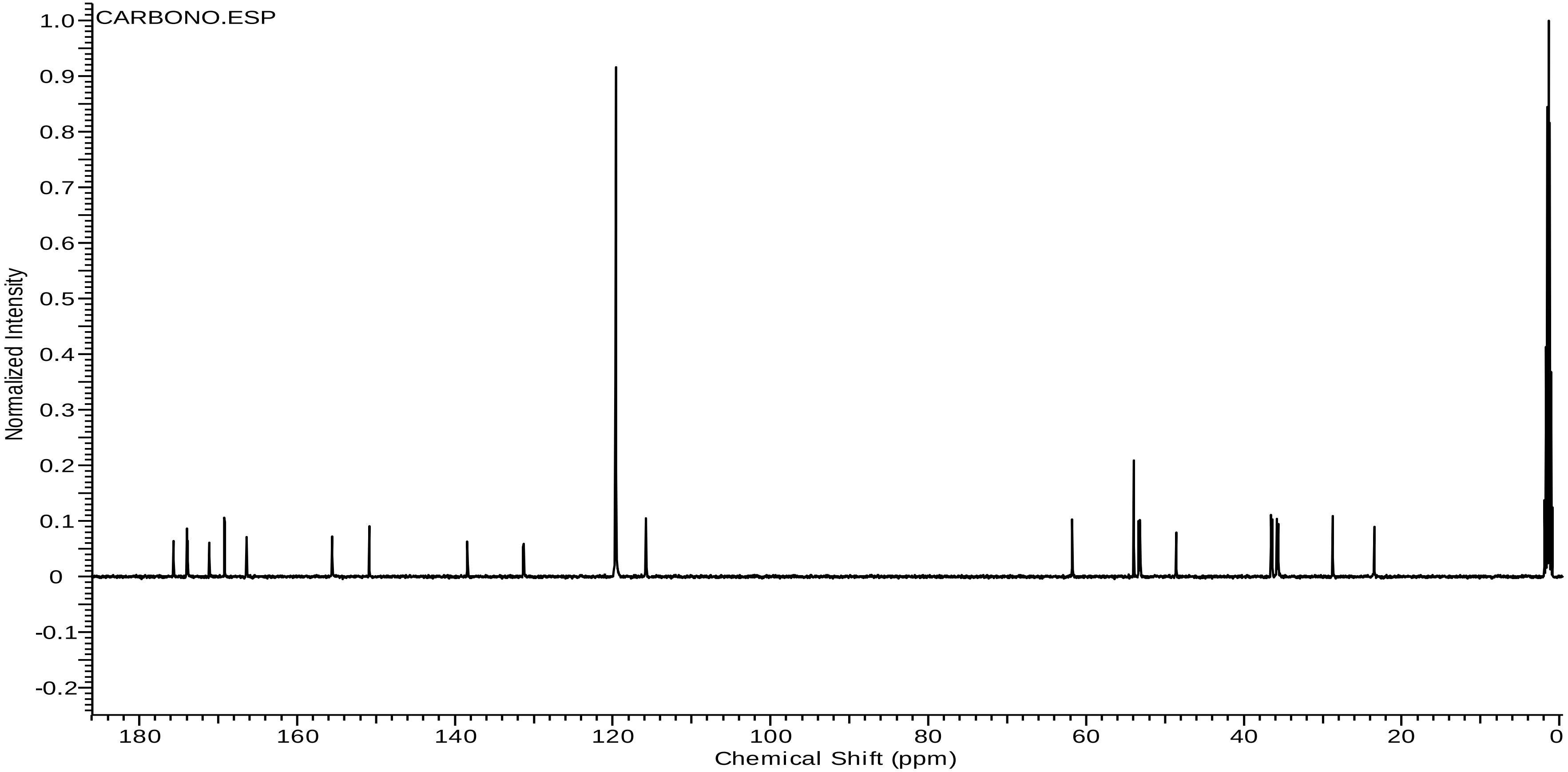

Figure S9. ^{13}C NMR spectrum of compound **1** (CD_3CN/D_2O 1:1).

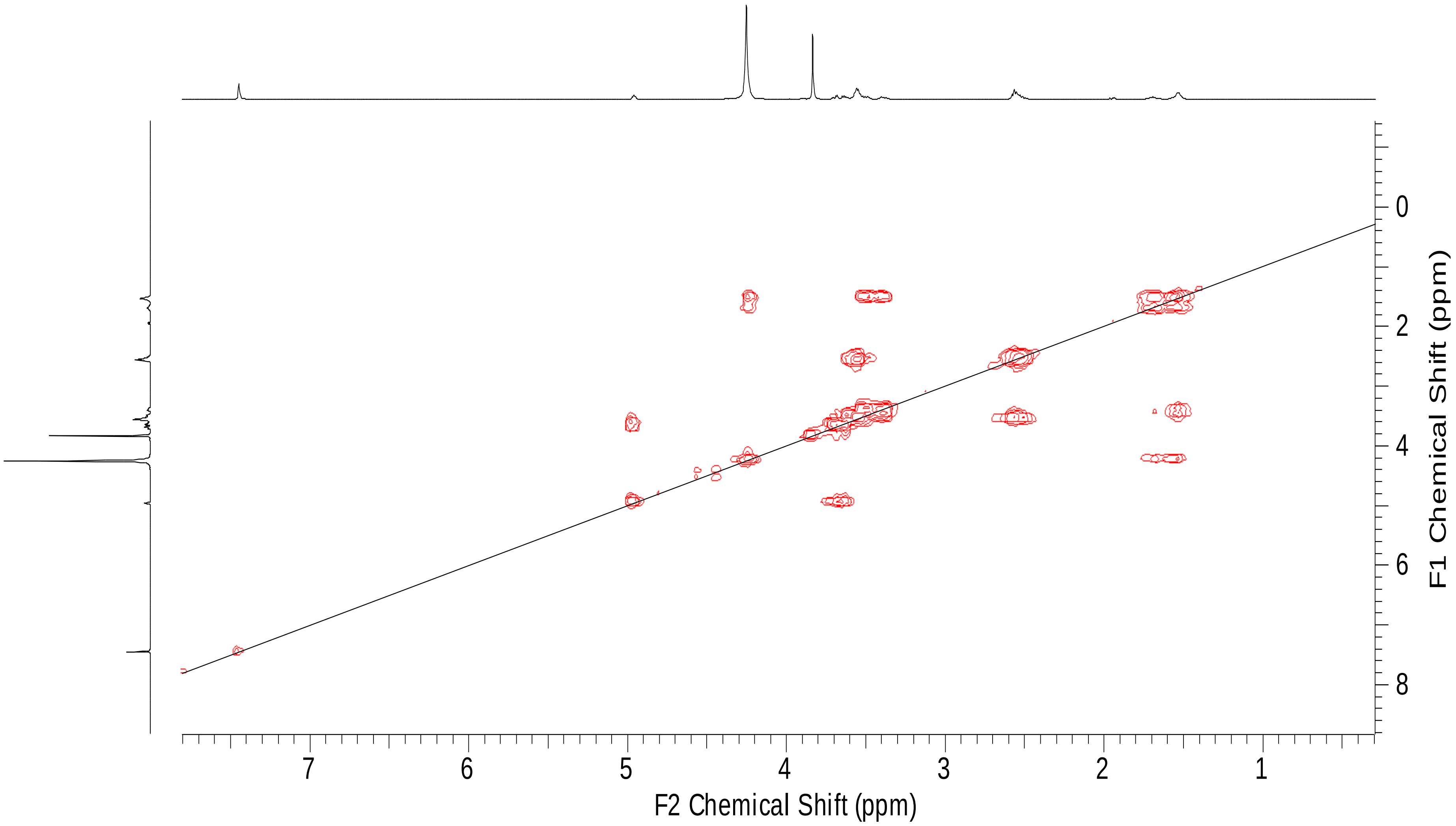

Figure S10. COSY spectrum of compound **1** (CD_3CN/D_2O 1:1).

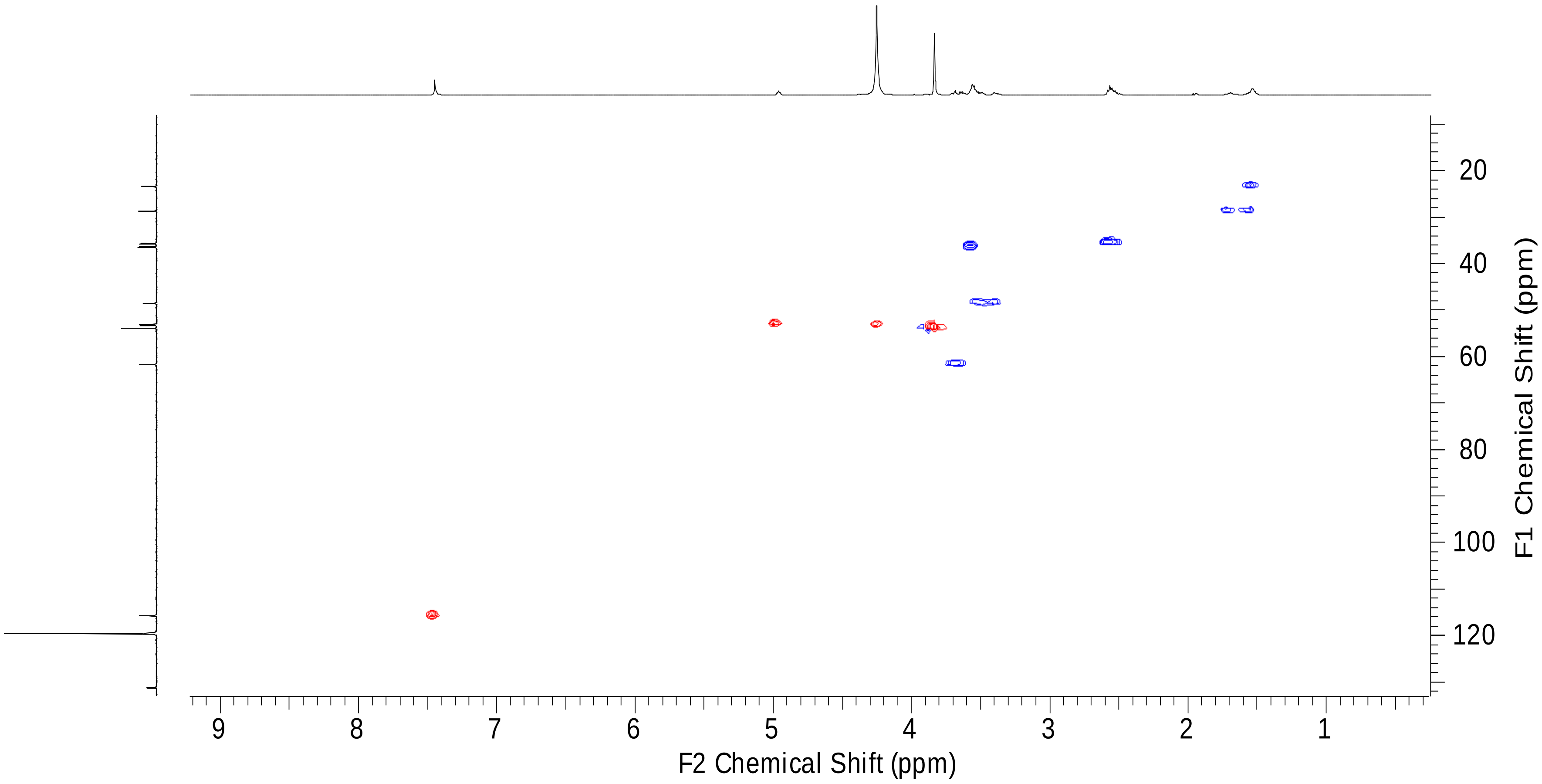

Figure S11. HSQC spectrum of compound **1** (CD_3CN/D_2O 1:1).

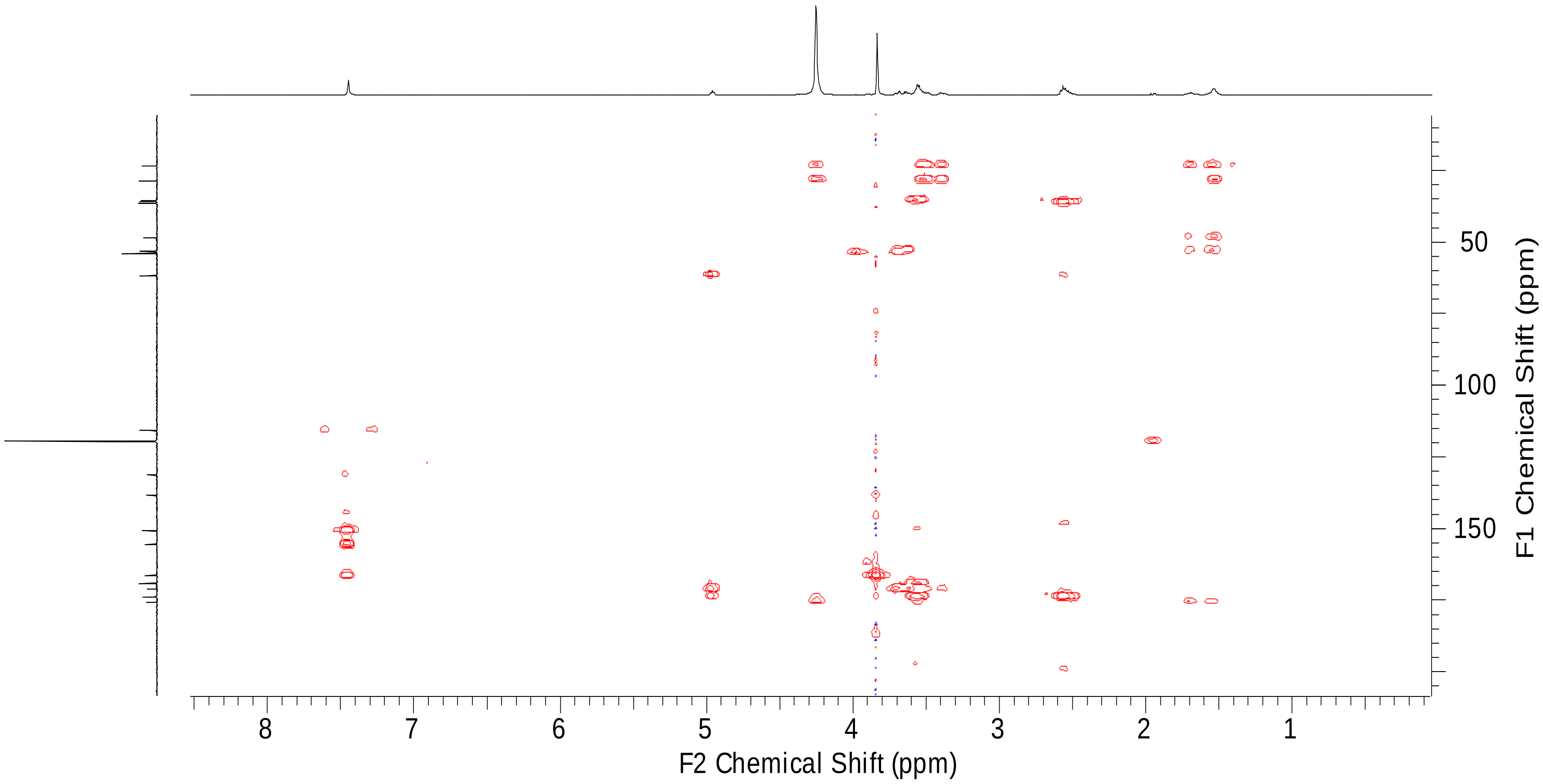

Figure S12. HMBC spectrum of compound **1** (CD_3CN/D_2O 1:1).

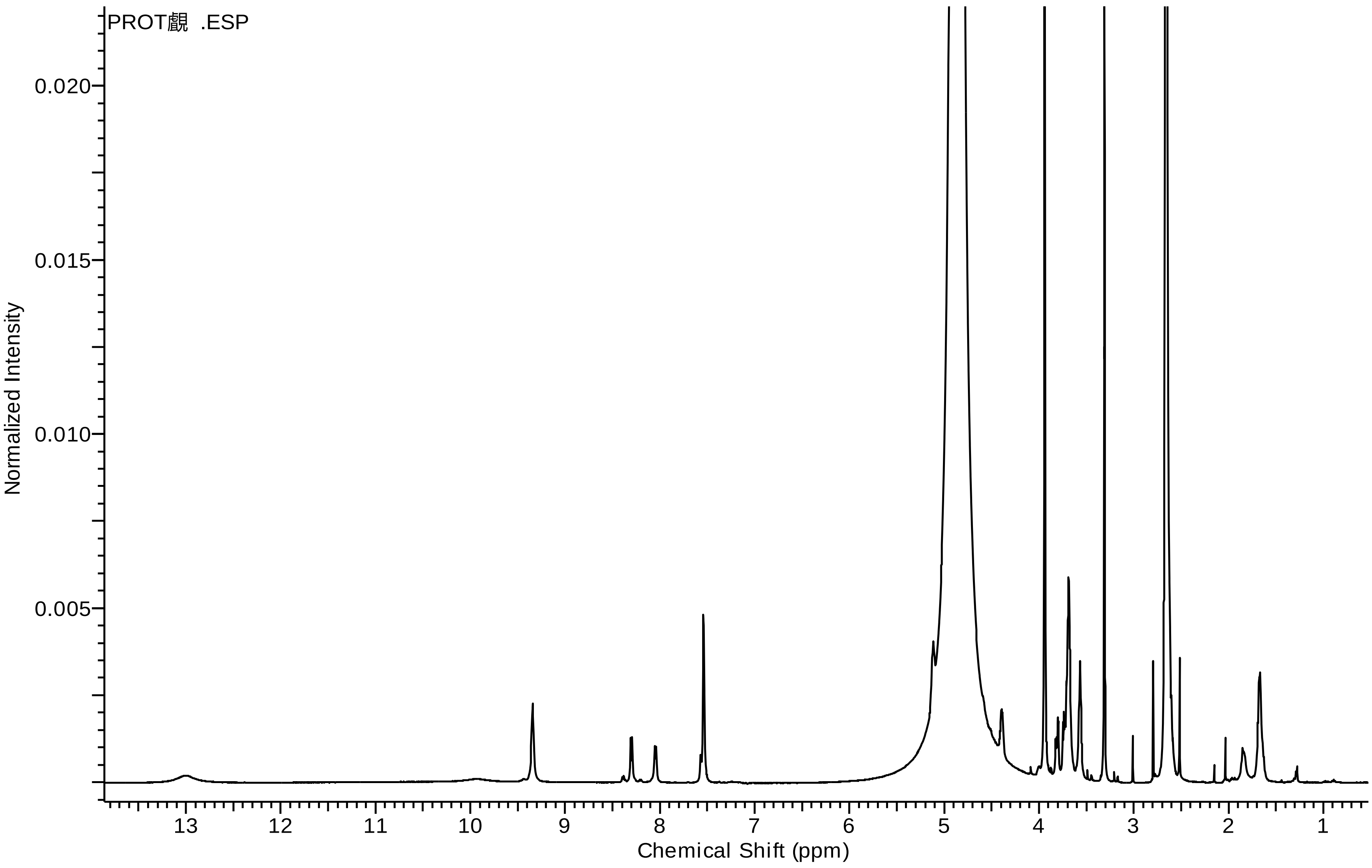

Figure S13. ^{1}H NMR spectrum of compound **1** (CD_3OH).

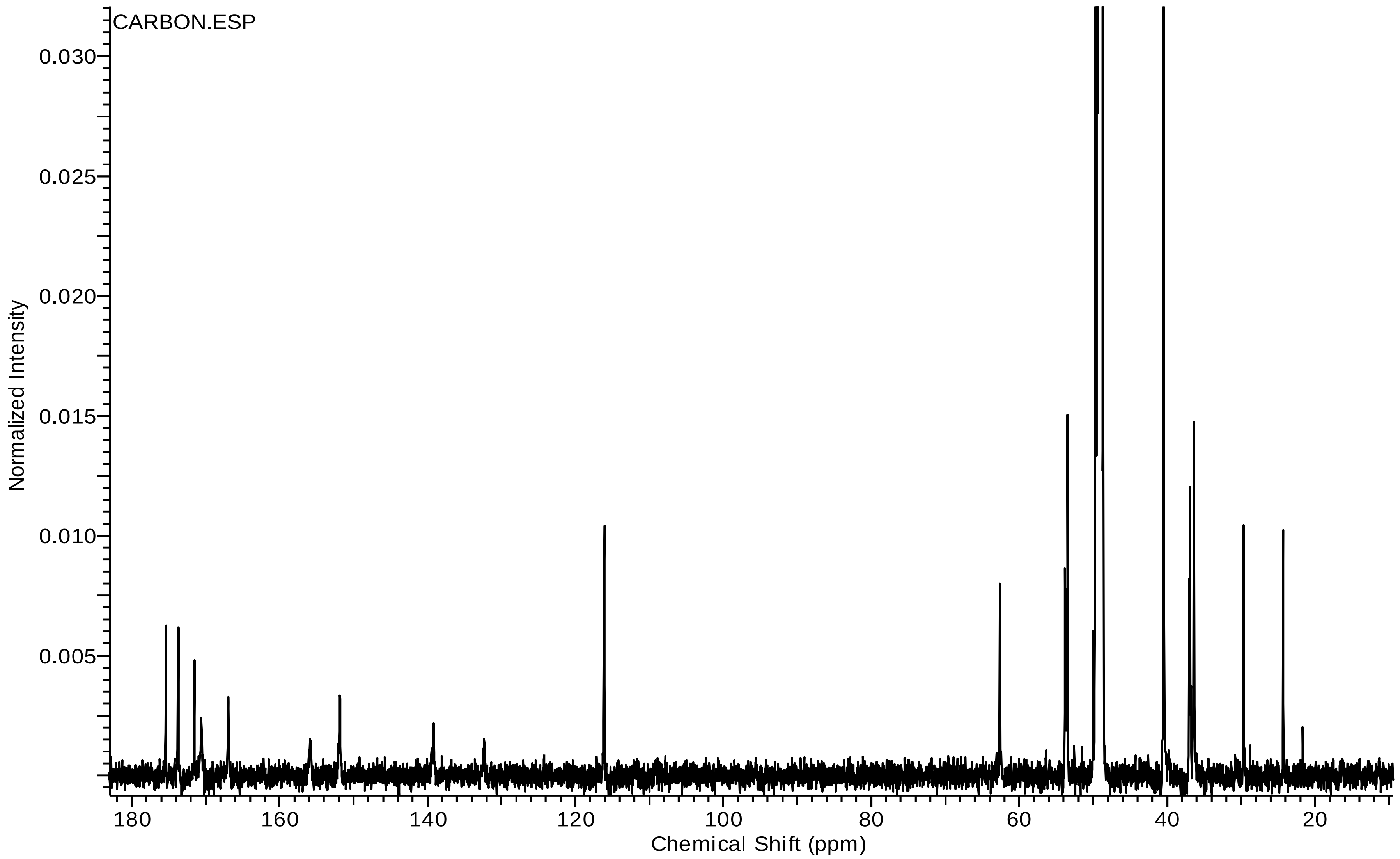

Figure S14. ^{13}C NMR spectrum of compound **1** (CD_3OH).

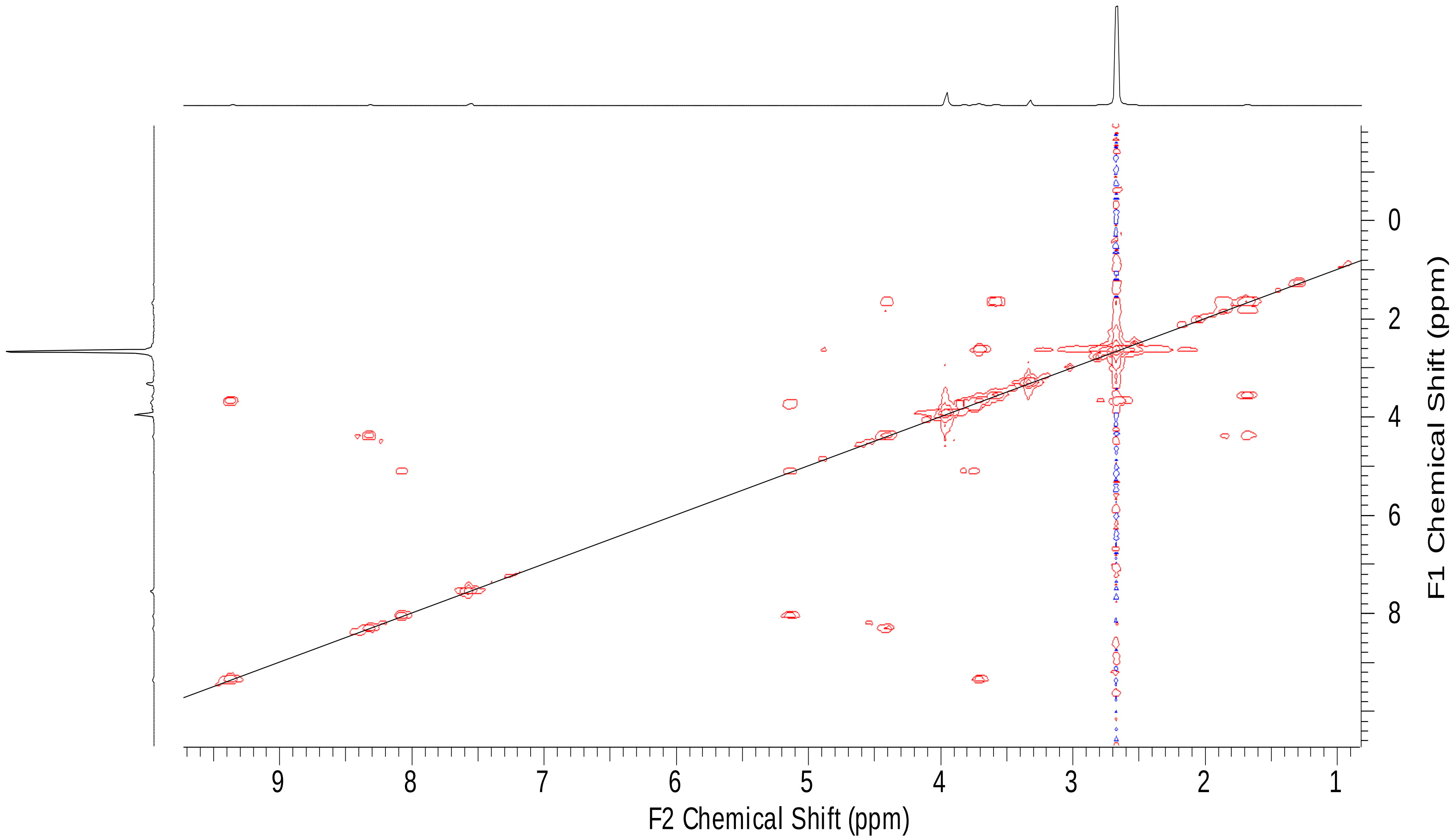

Figure S15. COSY spectrum of compound **1** (CD_3OH).

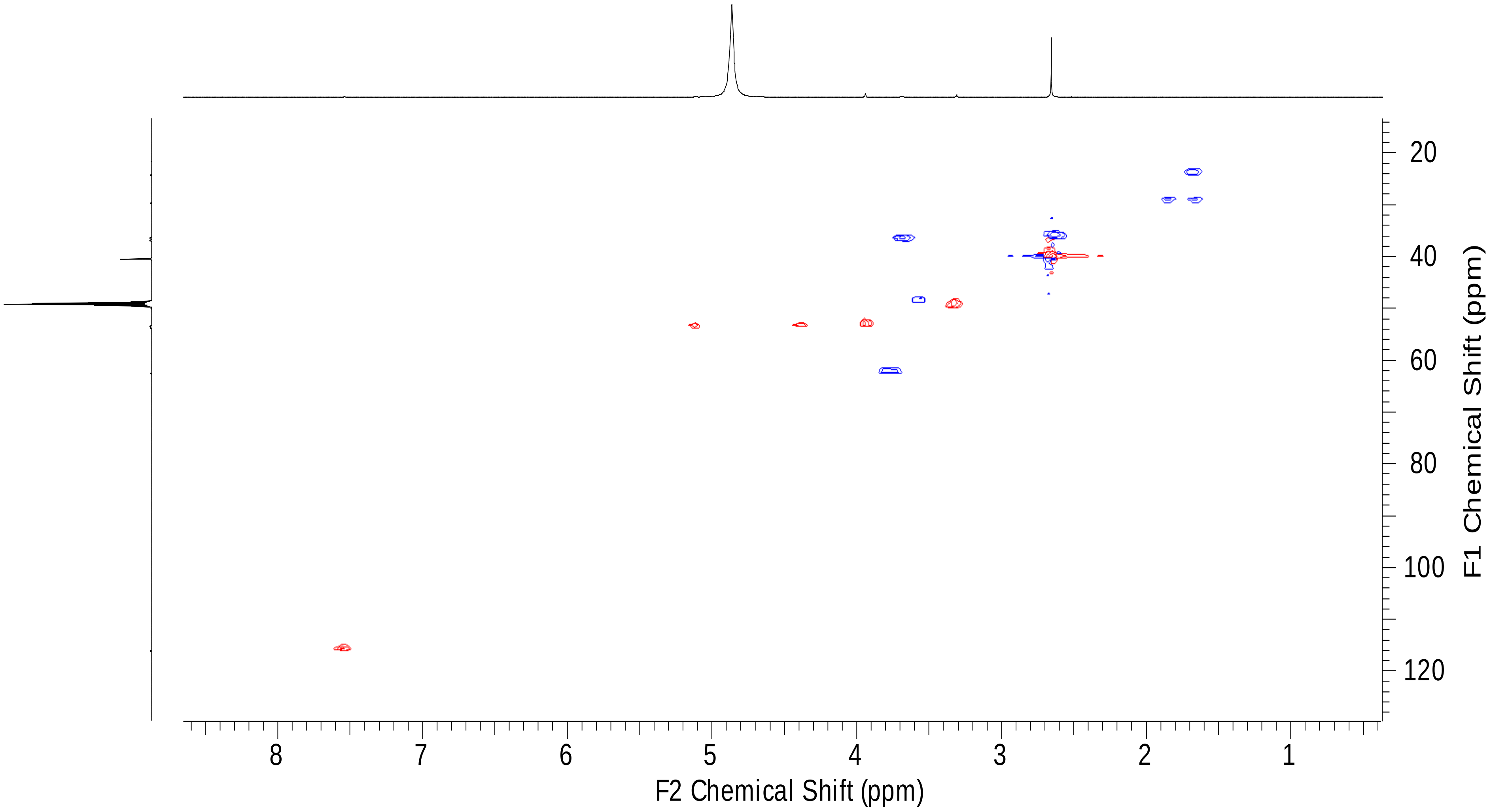

Figure S16. HSQC spectrum of compound **1** (CD_3OH).

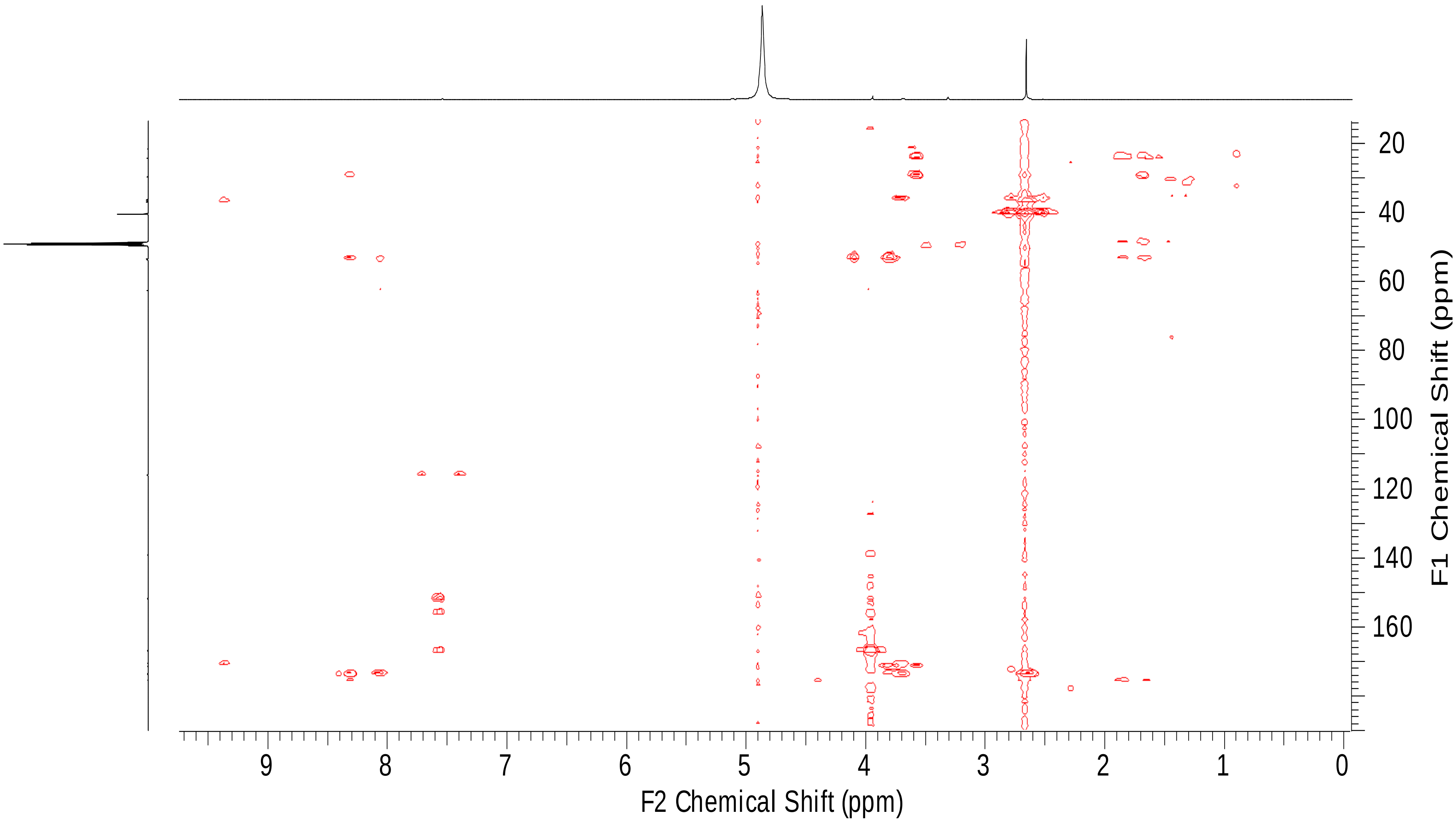

Figure S17. HMBC spectrum of compound **1** (CD_3OH).

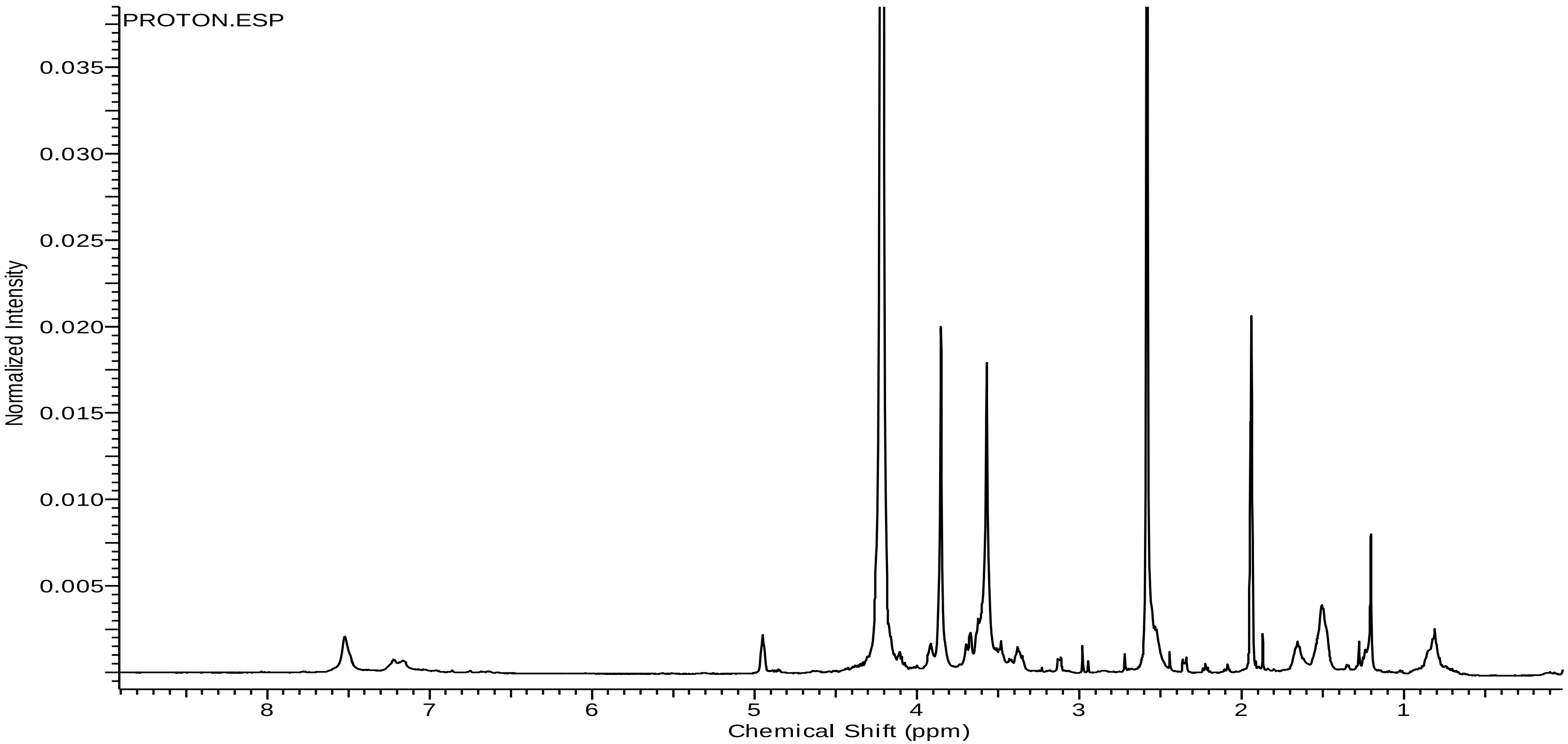

Figure S18. ^{1}H NMR spectrum of compound **2** (CD_3CN/D_2O 1:1).

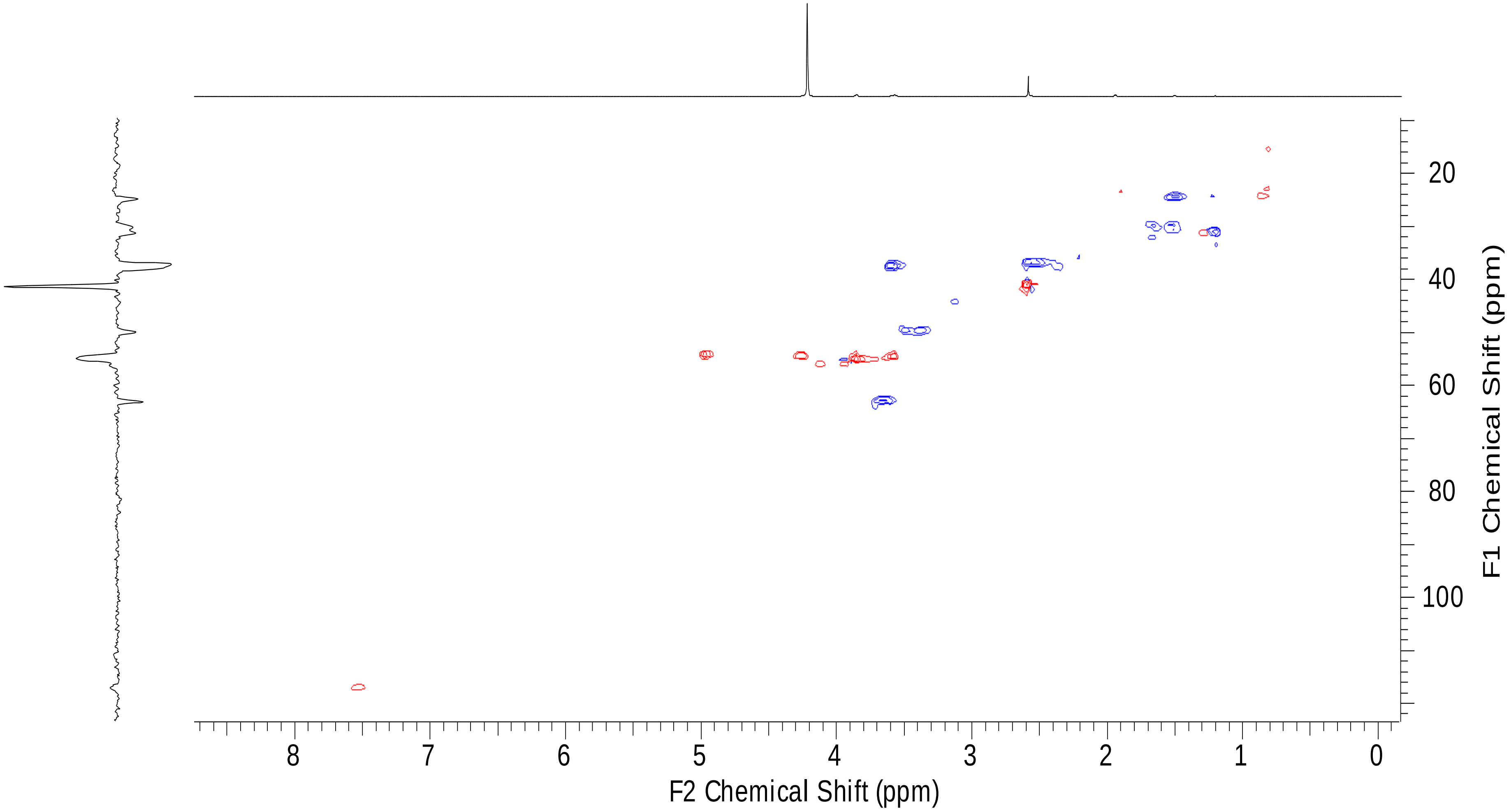

Figure S19. HSQC spectrum of compound **2** (CD_3CN/D_2O 1:1).

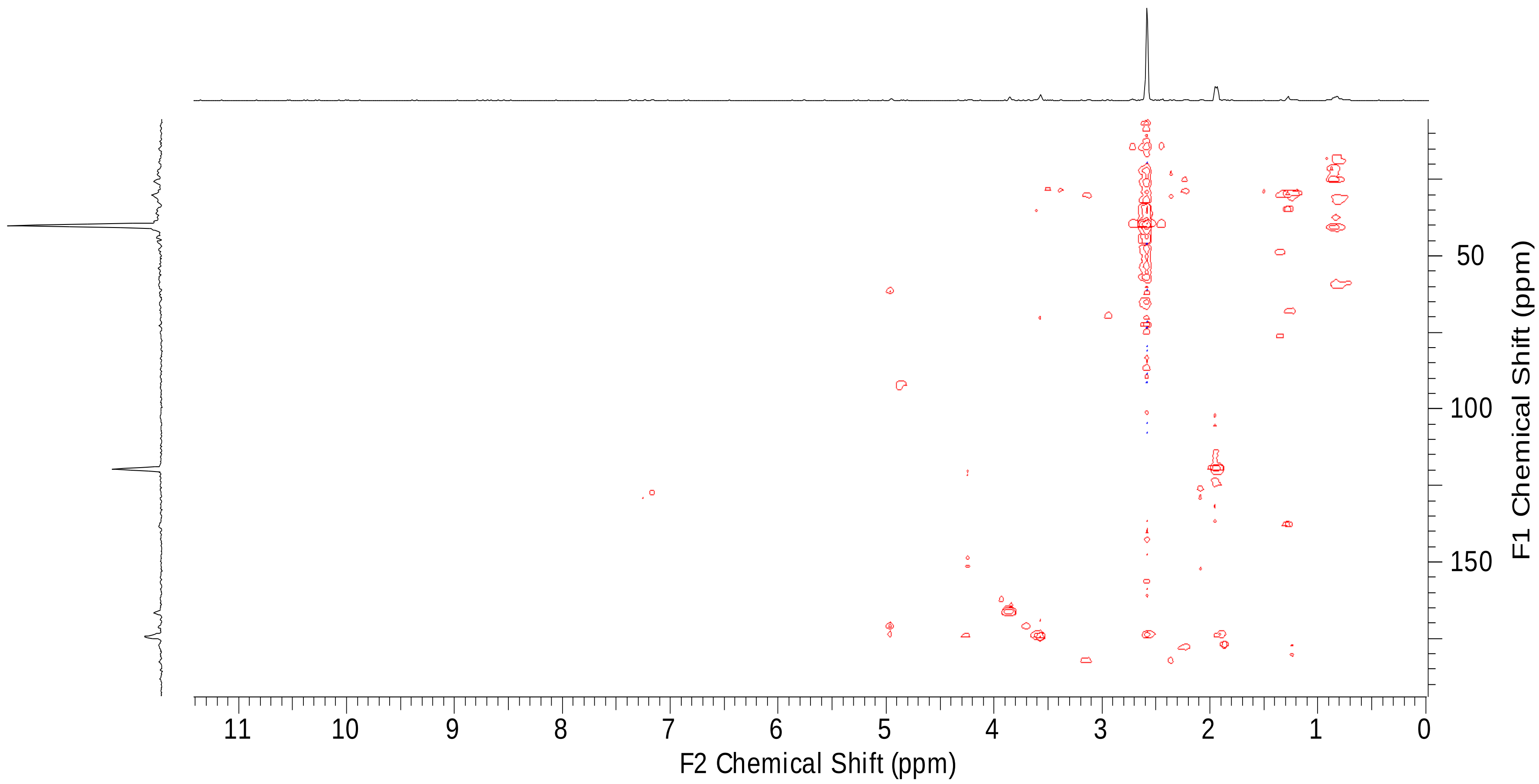

Figure S20. HMBC spectrum of compound **2** (CD_3CN/D_2O 1:1).

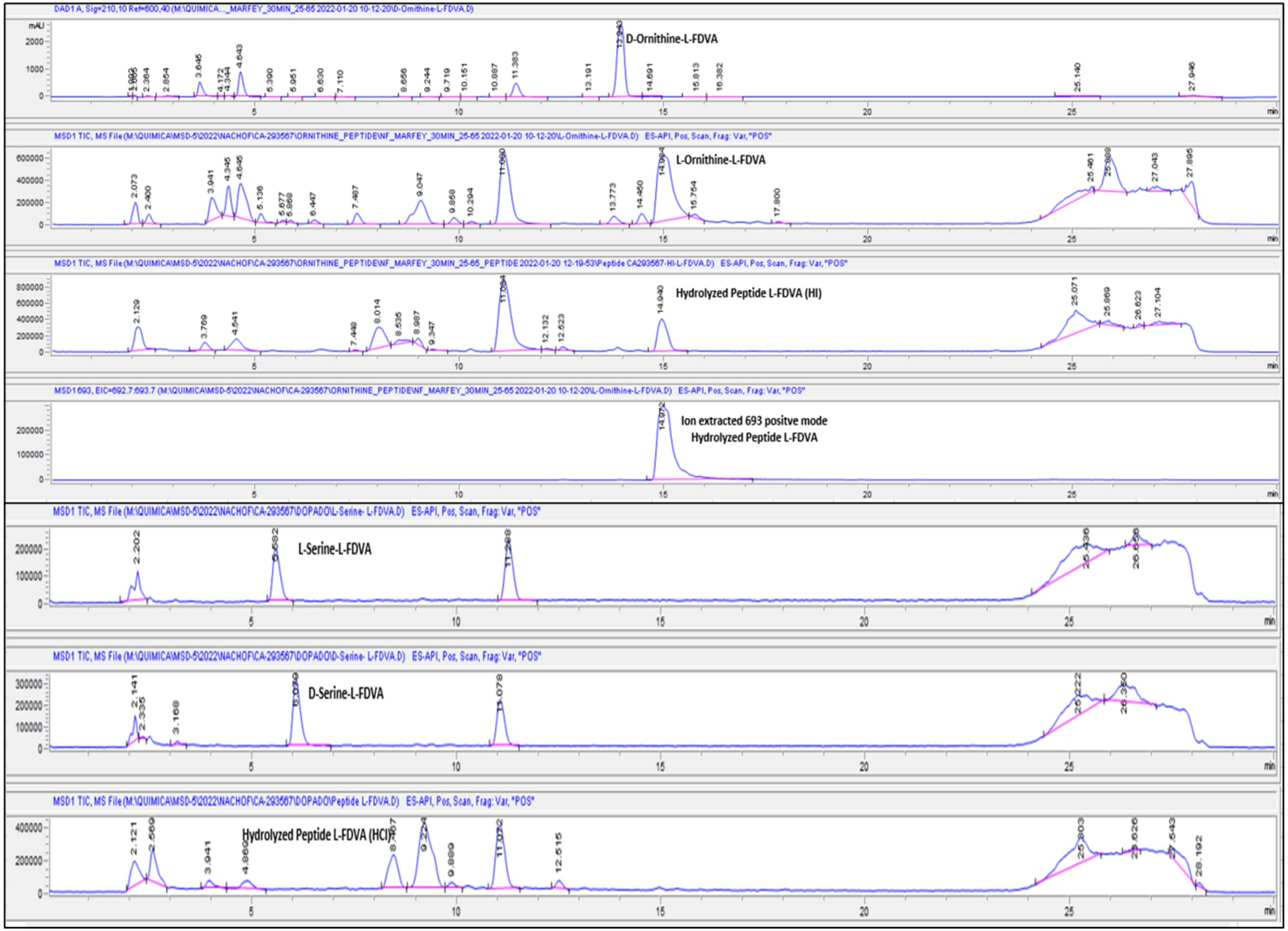

Figure S21. HPLC traces corresponding to Marfey's analysis of **1** (I).

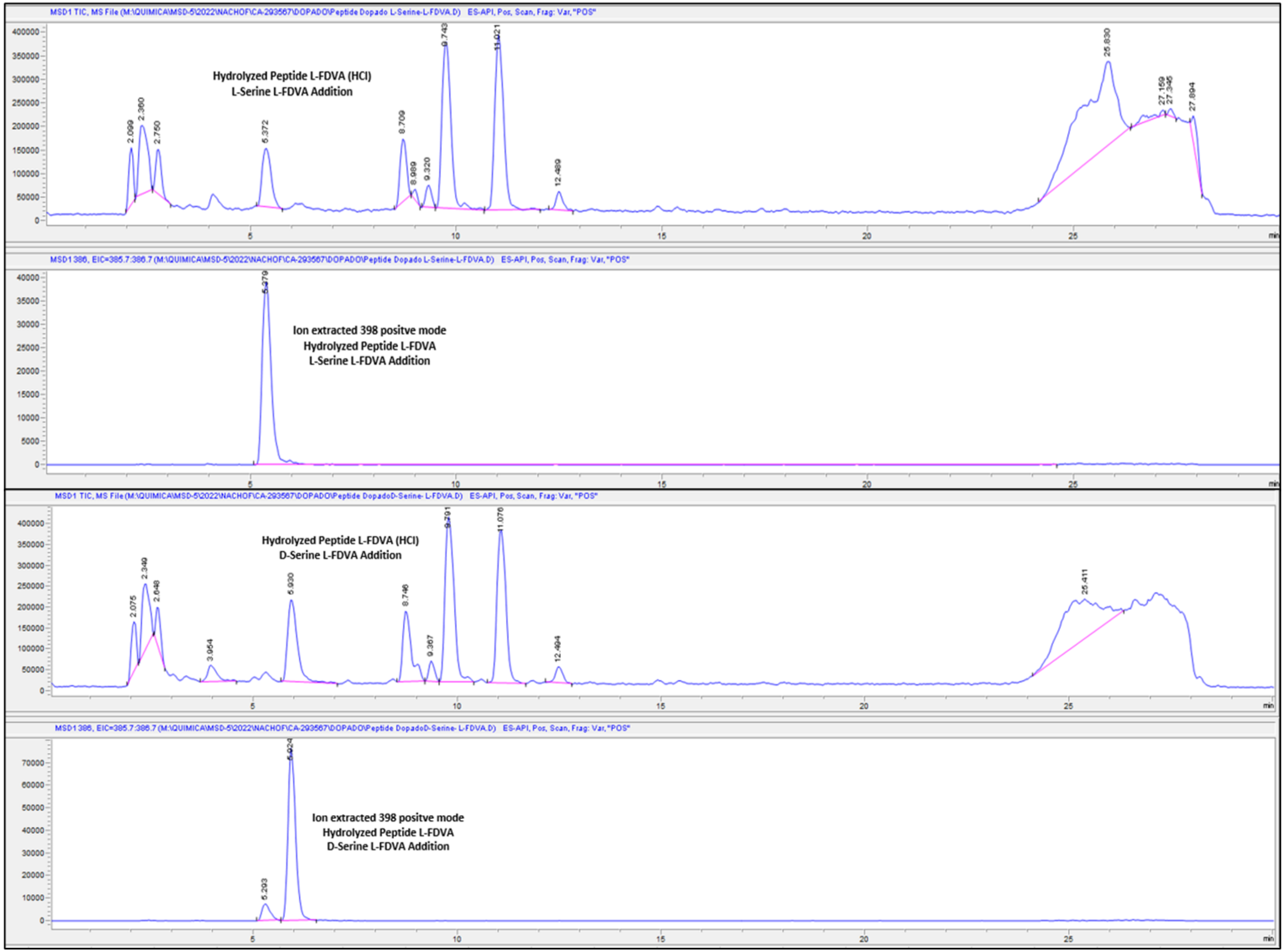

Figure S22. HPLC traces corresponding to Marfey's analysis of **1** (II).
